# Socioeconomic Inequalities in Mortality After Age 67: The Contribution of Psychological Factors

**DOI:** 10.3389/fpsyg.2021.717959

**Published:** 2021-10-06

**Authors:** Marijke Veenstra, Gøril Kvamme Løset, Svein Olav Daatland

**Affiliations:** NOVA, Oslo Metropolitan University, Oslo, Norway

**Keywords:** life span developmental theory, aging, socioeconomic inequalities, mortality, perceived control, subjective age

## Abstract

Diverging trends of longer lives and increased inequalities in age-at-death invite to updated research on late-life mortality. Earlier studies have identified health behavior, childhood, psychosocial, and material conditions as key determinants of life expectancy, but the role of psychological factors remains a topic of debate. The current study is framed in a life course developmental perspective and assesses the mediating role of secondary control strategies (subjective age) and primary control capacity (perceived control) to socioeconomic (wealth and education) inequality in mortality after age 67. Data are derived from the second wave of the Norwegian Life Course, Ageing and Generation study (*N*=1,432 and age 67–85). All in all, 366 deaths were observed over a mean follow-up of 9.6years. Perceived control was measured by the Pearlin and Schooler Mastery Scale. SA was measured with proportional discrepancy scores in felt age and ideal age. Stepwise Cox proportional hazards regression analyses were conducted to analyze the relative contribution of SES (education and accumulated wealth), felt age, and ideal age discrepancies and perceived control on 11-year mortality. Findings show that lower levels of wealth and perceived control independently predict increased elderly mortality over an 11-year period. Feeling younger and smaller ideal age discrepancies are positively associated with perceived control, but do not account for additional variability in longevity among older adults. Findings add to the interdisciplinary field of socioeconomic inequalities in elderly mortality and underline the specific importance of structural conditions (wealth) and the continued importance of (perceived) primary control capacity for longevity also after age 67. Future research may assess in more detail how wealth and perceived control complement each other in contributing to healthy aging and longevity, for example, by longitudinal research including the role of significant life events in the second half of life in different welfare state contexts.

## Introduction

Longevity increases, and larger parts of the population, in particular those with higher socioeconomic status (SES), survive to more advanced ages (Murtin et al., 2017; OECD, 2017; Kinge et al., 2019; Permanyer and Scholl, 2019). The resulting diverging trends of longer lives and increased inequalities in age-at-death have prompted recent calls for more research on factors contributing to patterns of elderly mortality, that is, patterns conditional upon survival to, for example, the standard retirement age (Abramson and Portacolone, 2017; Permanyer and Scholl, 2019; Aburto et al., 2020). Extensive research has identified childhood and material living conditions as well as behavioral and psychosocial characteristics as core determinants of (inequalities in) life expectancy (Mackenbach et al., 2019). However, the contribution of psychological factors to life span variability among older adults is still a topic of debate. Over the life course, differences in SES render differences in material and coping resources (e.g., beneficial social connections and control), thereby contributing to differential exposure to stressors and to differential vulnerability (Phelan et al., 2010). In old age, individuals from divergent backgrounds face life transitions that typically constitute moments of vulnerability, that is, declining mobility, onset of health problems, and the loss of close family members and friends (Heckhausen, 1997; Abramson and Portacolone, 2017). Examining the different, and sometimes similar, perceptions of aging and adaptation in older people may provide a powerful vantage point for understanding how psychological factors shape inequalities in elderly mortality. The current study departs from life span developmental theory (LSD; Heckhausen, 1997; Heckhausen et al., 2019), which assumes that people actively shape their own development, and examines the contribution of psychological factors, that is, perceived control and subjective age perceptions, for mortality patterns in adults aged 67 and older.

The basic assumption of LSD theory is that “individuals in general strive to maximize primary control of their environment and developmental outcomes by adapting their regulatory strategies to the opportunities and constraints encountered in a given situation” (Heckhausen et al., 2019). In this way, older adults do not qualitatively differ from younger adults but they have to strive for control in a life phase that is characterized by developmental losses more than by gains (Heckhausen et al., 2010) and where “control-enhancing experiences” get rarer and “control-restricting circumstances” increase (Wolinsky et al., 2003). This means that although *striving* for primary control is assumed to remain constant throughout the life course, the *capacity* for primary control, often proxied by measures of perceived control, tends to gradually decline in old age (Slagsvold and Sørensen, 2013; Robinson and Lachman, 2017; Heckhausen et al., 2019). Perceived control over life circumstances is also inversely distributed by social status: those with higher education or income perceive greater control (Elstad, 1998; Mirowsky and Ross, 2007). Moreover, the less advantaged are found to face declining primary control capacity earlier in life than those from more advantaged positions (Heckhausen et al., 2010). So far, some empirical evidence suggests that low socioeconomic status is related to mortality partly because people with a low socioeconomic status more often perceive low control (Bosma et al., 1999) and that those with a lower SES who do have higher levels of perceived control tend to have health and longevity similar to those with a higher SES (Lachman and Weaver, 1998; Turiano et al., 2014; Robinson and Lachman, 2017).

Higher SES is associated with greater access to resources throughout the life course, which in turn facilitates regulation of developmental trajectories, including analyzing opportunities to optimize goal choices to ensure higher control capacity. Older individuals from advantaged SES backgrounds may have multiple goal-path options to strengthen control capacity (Heckhausen et al., 2010), whereas disadvantaged individuals may have few. These different goal-path options can be grouped into primary and secondary control strategies. Primary control strategies aim at “changing the external environment in line with one’s wishes,” whereas secondary control processes imply “changing the self to bring oneself in line with environmental forces” (Heckhausen et al., 2019). The use of secondary control strategies, that is, selective and compensatory secondary control, tends to become more common in old age (Schulz and Heckhausen, 1996; Thompson et al., 1998; Chipperfield et al., 1999). Selective secondary control serves to enhance and maintain commitment to a feasible goal (Heckhausen et al., 2010), for example, through positive illusions about one’s control potential for achieving the goal. Compensatory secondary control strategies are aimed at minimizing negative effects of loss on the individual’s perceived control (Heckhausen, 1997), which can be achieved by, for example, goal disengagement or strategic social comparison (Bailis et al., 2005; Heckhausen et al., 2010).

A potentially important secondary control strategy to face challenges associated with advanced age is subjective age identification (Heckhausen, 1997; Shane et al., 2019) – the way individuals think about age and the stages of life they are in. Subjective age (SA) is a core indicator of the individual aging experience, with important anticipated consequences for successful aging in older adults (Kastenbaum et al., 1972; Kleinspehn-Ammerlahn et al., 2008; Kotter-Gruhn et al., 2016). SA comprises different components, including felt age and ideal age (Kastenbaum et al., 1972; Uotinen et al., 2005; Veenstra et al., 2020). Research on SA can be traced back to as far as the 1950s (Blau, 1956) and has since then consistently shown that from early middle age onwards, an increasing proportion of people feels younger than their actual age, and would like to be even younger (Barak and Stern, 1986; Montepare and Lachman, 1989; Hubley and Hultsci, 1994; Daatland, 2007). SA is therefore assumed to gain particular importance in the second half of life, where attitudes toward aging are assumed to become more self-relevant than actual age (Settersten and Mayer, 1997; Diehl et al., 2014; Kornadt et al., 2018). SA is strongly affected by age stereotypes (Levy, 2009). As people grow older, the stereotype age norms prevailing in society may make it difficult to adopt an older identity without losing a sense of continuity of the self as they are confronted with age-related physical, cognitive, and social changes (Westerhof et al., 2012; Settersten and Hagestad, 2015). Older adults who report feeling younger than their chronological age are using a selective secondary control strategy that enables a feeling of continuation of one’s self-perception. This, in turn, may enhance vitality and resourcefulness, which increases overall perceived control capacity and encourages selective primary control striving. Empirical findings support this assumption (Shane et al., 2019) and have also shown that feeling younger is associated with reduced mortality (Uotinen et al., 2005; Kotter-Gruhn et al., 2009; Stephan et al., 2018).

Wanting to be younger than one’s actual age (younger ideal age) constitutes a compensatory secondary control strategy aimed at identifying with an age that reflects desired control potential (Heckhausen, 1997). It is not uncommon for older adults to report an ideal age which is 18 to 19years younger than one’s current age (Uotinen et al., 2006; Keyes and Westerhof, 2012; Veenstra et al., 2020). However, findings from previous studies consistently show that wanting to be increasingly younger is associated with poorer health outcomes, including higher mortality (Maier and Smith, 1999; Uotinen et al., 2006; Keyes and Westerhof, 2012; Veenstra et al., 2020). A large discrepancy between ideal age and one’s chronological age may arise when there is an incongruence between the aspired goal and actual constraints in opportunities to reach this goal and reflects “withdrawing effort without breaking up the motivational commitment” (Wrosch et al., 2003), “a passive reflection of failure and loss” (Heckhausen et al., 2010), and feelings of regret (Heckhausen et al., 2019). On the other hand, an ideal age close to one’s actual age reflects more positive self-perceptions of aging and higher age satisfaction (Hubley and Hultsci, 1994; Kaufman and Elder, 2002). An earlier study by Levy et al. (2002) showed that individuals with positive self-perceptions of aging lived on average 7.5years longer than individuals who had negative self-perceptions.

In sum, whereas feeling younger may serve as an adaptive selective secondary control strategy in response to aging and thus contribute to perceived control and longevity (Westerhof et al., 2014), wanting to be younger may function as a maladaptive compensatory secondary strategy (Keyes and Westerhof, 2012) associated with perceived lower control and increased mortality. SES is considered a core determinant of subjective aging through its influences on the timing of life transitions and rate of health decline (Barrett, 2003). Consequently, those with lower SES tend to adopt older age identities and to show less satisfaction with aging (Kleinspehn-Ammerlahn et al., 2008).

The present study extends existing knowledge by assessing the relative contribution of perceived control and domains of SA to socioeconomic inequalities in elderly mortality. More specifically, we argue that subjective age perceptions are important secondary control strategies for primary control capacity (perceived control), which both are influenced by levels of SES and, this way, mediate the association of SES with longevity in older adults.

## Materials and Methods

### Study Procedure and Participants

We used data from the second wave of the longitudinal Norwegian Life course, Ageing and Generation study (NorLAG), which collects data from a nationwide, population-based, and stratified sample of adults born between 1922 and 1966 (Veenstra et al., 2021). NorLAG combines survey data linked to data from national public registers. The second wave (NorLAG2) was conducted in 2007–2008, and the full sample includes 9,238 respondents (response rate 61%), of whom 1,673 persons are 67years and older. Survey data collection consisted of an initial telephone interview followed by a postal questionnaire. As is common in most longitudinal studies, selective attrition is a challenge also in NorLAG: Respondents with higher education and good self-rated health are more inclined to participate. We refer to NorLAG’s cohort profile (Veenstra et al., 2021) for more detailed information about design, samples, and response rates. Study participation and linkages to register data are based on informed consent. All participants are listed in the Population Register that provides information on all-cause mortality up to the start of the data collection for the third wave of NorLAG (March 31st, 2017). The analyses in the current study are based on complete data, including respondents who returned the postal questionnaire and had valid answers on SA and perceived control (*N*=1,432). Compared to those who only participated in the telephone interview, those who returned the postal questionnaire were higher educated (*χ*^2^=17.4; *df*=2; *p*<0.001), somewhat younger (*t*=3.10; *p*=0.002), somewhat more likely to have good/excellent self-rated health (*χ*^2^=4.2; *df*=1; *p*=0.041), and to be female (*χ*^2^=3.9; *df*=1; *p*=0.049). Mortality rates did not differ significantly between these two groups (*χ*^2^=1.9; *df*=1; *p*<0.158).

### Measures

#### Survival Time

We measure survival time as year of baseline interview in NorLAG2 (2007 or 2008) to the year of either death or end of follow-up (March 2017). Ninety-five percent of the sample was interviewed in 2007. Due to reasons of confidentiality, only the year of death was included in NorLAG. Two respondents died during the same year they were interviewed. Survival time for these persons was set to 0.5.

#### Socioeconomic Status

SES is assessed by educational attainment and accumulated wealth at the time of the interview. Level of education was derived from the public registries (Norwegian Standard Classification of Education Revised 2000) and grouped into three categories: compulsory (1), secondary (2), and tertiary education (3). Data on taxable gross wealth are available through the linkages with the tax register and comprises taxable real capital and taxable gross financial capital rounded to the nearest 10,000 NOK. Wealth data were grouped into quartiles <280,000 (1); 290,000–550,000 (2); 551,000 – 1,070,000 (3); and>1,070,000 (4).

#### Perceived Control

Perceived control is a assumed to be a proxy for (subjective) control capacity and is measured in NorLAG2 through the self-administered questionnaire with the Pearlin and Schooler’s Personal Mastery Scale (PMS; Pearlin and Schooler, 1978). The PMS measures the self-belief or conviction that one is able to control the important circumstances that are currently impinging on one’s life (Pearlin and Schooler, 1978; Pearlin et al., 1981). The PMS consists of seven items, of which two are positively phrased. Examples of items are as follows: “I have little control over the things that happen to me”; “I can do just about anything I really set my mind to”; “There is really no way I can solve some of the problems I have”; and “What happens to me in the future mostly depends on me.” Responses are measured on a 5-point Likert scale (1 “fully agree” and 5 “fully disagree”), which are recoded and aggregated into a single total sum score, ranging from 5 to 35, with higher scores indicating stronger feelings of sense of control. Cronbach’s alpha for the PMS in the current study was 0.70 indicating satisfactory reliability.

#### Subjective Age (SA)

The items measuring felt age and ideal age are part of the postal questionnaire. Felt age is assessed with the item: “What age do you usually feel?” Ideal age is measured by asking: “If you could choose your age, what age would you like to be?” In both instances, respondents wrote down the age in number of years, which provides information on the direction as well as the magnitude of the subjective age discrepancy. Information on respondents’ chronological age at time of interview is derived from the public registers and linked to the survey data. We calculated the proportional discrepancy score by subtracting felt age from chronological age (chronological age – felt age) and divide this by chronological age. Proportional discrepancy scores for ideal age are calculated in the same manner. A negative value thus indicates older subjective ages, whereas positive values indicate that a person feels (felt age) or wants to be (ideal age) younger than his or her chronological age.

#### Confounders

In addition to age at time of baseline interview and gender (0=male; 1=female), we control for physical health limitations (0=no; 1=yes), living with a partner/spouse (0=no; 1=yes), and smoking cigarettes (0=no; 1=yes). All confounders refer to baseline measures in NorLAG2. Adjusting for these variables is necessary because they have known associations with SES and perceived control, as well as mortality (Mackenbach et al., 2019).

### Statistical Analyses

We use summary statistics (*t* tests, chi-square statistics) and bivariate correlations (Pearson’s r) to describe the central study variables and their relationships. Associations of baseline measures with mortality endpoints are assessed using stepwise Cox proportional hazards regression. Hazard ratios (HR) and their 95% confidence interval (CI) are used to summarize the associations. We compute standardized scores (Z-scores) for the PMS and the two domains of SA. This way, coefficients can be interpreted as change in standard deviations (SD). We start with computing the unadjusted (bivariate) HRs for the core variables of interest. The first model (Model 1) in the stepwise Cox proportional hazards regression is the baseline model with the basic demographic variables: age and gender. The second model adds markers of SES: wealth and level of education. In the third model, we also include the two measures of SA (felt age and ideal age discrepancies) and in the fourth model perceived control as measured by the PMS is added. The full model (Model 5) includes other known risk factors (health, partner status, and health behavior) as well. To estimate how much the different steps contributed to explained variance, we use the generalized R-squared, which produces a pseudo-R-squared, calculated as 1-exp[(*χ*LR2)/n], where (*χ*LR2) is the chi-square statistic for the likelihood ratio test for the model, and n is the total number of cases. For all statistical tests, we apply a critical value (ɑ) of 5%.

As a robustness check, we conducted similar analyses with logistic regression models with survival status as dichotomous outcome, thereby ignoring the information on time to death. These analyses (available upon request) yielded similar results, and we therefore only report the findings from the Cox regression analyses.

## Results

During a mean follow-up time of 9.6 (*SD*=2.7) years, 366 deaths from all causes occurred, corresponding to 25.6 percent of the sample of persons aged 67years and older. A description of the characteristics of the full sample and across mortality status is given in Table 1.One out of four of the respondents had tertiary (higher) education, but a significantly greater share of those who died over the follow-up were in the group with primary education (28.1 percent). Similarly, those who had died were less likely to be from the highest wealth quartile (19.7 percent) compared to those who were alive at follow-up (26.7 percent). Average perceived control as measured by the PMS was significantly lower among those who had died (23.0 versus 25.1). Proportional differences scores for felt age and ideal age were 0.12 and 0.33, respectively. This implies that, on average, individuals felt 12 percent younger and had an ideal age which was 33 percent younger relative to their chronological age. People who died felt on average 8.2years younger than their actual age, compared to 9.4years among those who survived. These differences were statistically significant and corresponded to proportional discrepancy scores of 0.11 and 0.13. Moreover, people who had died during follow-up had, on average, an ideal age that was 26.9years younger than their chronological age, which corresponded to a proportional discrepancy score of 0.36. Those who survived had an average ideal age discrepancy of 23.7, corresponding to a proportional discrepancy score of 0.33. These differences in ideal age discrepancies across mortality status were statistically significant as well. In addition, those who died during follow-up were more likely to be men (61.5 percent), have physical health problems (37.7 percent), smoke cigarettes (21.0 percent), and less likely to live with a partner at baseline (59.3 percent).

**Table 1:** Baseline characteristics of the study sample according to survival status at the end of follow-up.

Variables	People who survived	Deceased	Total sample
*N*	*1,066*	*366*	*1,432*
Women (%)^***^	52.3	38.5	48.7
Chronological age, mean (SD)^***^	72.2 (4.3)	75.5 (5.1)	73.1 (4.7)
Education (%)^*^			
Primary	22.8	28.1	24.2
Secondary	50.2	51.6	50.6
Tertiary	27.0	20.2	25.3
Wealth in NOK - quartiles^*^			
< 280,000	24.5	27.6	25.3
290,000–550,000	23.7	27.3	24.7
560,000–1,070,000	25.0	25.4	25.1
> 1,070,000	26.7	19.7	24.9
Perceived control, mean (SD)^***^	25.1 (5.0)	23.0 (5.3)	24.6 (5.1)
Felt age discrepancy, mean (SD)^*^	9.4 (8.8)	8.2 (9.2)	9.1 (9.0)
Ideal age discrepancy, mean (SD)^**^	23.7 (16.2)	26.9 (17.7)	24.5 (16.7)
Felt age proportional discrepancy, mean (SD)^***^	0.13 (0.12)	0.11 (0.12)	0.12 (0.12)
Ideal age proportional discrepancy, mean (SD)^***^	0.33 (0.22)	0.36 (0.23)	0.33 (0.22)
Physical health problems (%)^***^	20.6	37.7	25.0
Living with a partner (%)^**^	67.9	59.3	65.7
Smoking cigarettes (%)^**^	14.1	21.0	15.9

* *p*<0.05;

** *p*<0.01;

*** *p*<0.001.

Table 2 provides an overview of the bivariate correlations between the study variables. Mortality was statistically significantly associated with all study variables. Strongest correlations were with chronological age (*r*=0.30), and the weakest correlations were with proportional discrepancy scores for ideal age (*r*=0.06). SES was significantly, although weakly, correlated with mortality, with *r*=0.08 and *r*=0.07 for educational attainment and wealth, respectively. The positive correlation of ideal age discrepancy scores with mortality implies that wanting to be increasingly younger is associated with higher mortality, whereas the negative correlation of felt age discrepancy scores with mortality indicates that feeling younger than one’s actual age is associated with reduced mortality. As anticipated, a perceived higher control was associated with less mortality (*r*=−0.18).

**Table 2:** Bivariate correlations (Pearson’s r) between the study variables (*N*=1,432).

Variables	1	2	3	4	5	6	7	8	9	10	11
1. Chronological age	1										
2. Perceived control	−0.21^**^	1									
3. Felt age proportional discrepancy	−0.08^**^	0.12^**^	1								
4. Ideal age proportional discrepancy	0.08^**^	−0.08^**^	0.18^**^	1							
5. Education (1–3)	−0.01	0.11^**^	0.00	−0.06^*^	1						
6. Wealth (1–4)	0.05	0.08^**^	−0.04	0.01	0.31^**^	1					
7. Gender (0=Man; 1=Woman)	−0.00	−0.09^**^	0.01	−0.19^**^	−0.12^**^	−0.24^**^	1				
8. Physical health problems (0=No; 1=Yes)	0.11^**^	−0.20^**^	−0.07^**^	0.04	−0.05	−0.11^**^	0.12^**^	1			
9. Cigarette smoking (0=No; 1=Yes)	−0.14^**^	−0.01	−0.05	0.03	−0.09^**^	−0.06^*^	0.03	0.01	1		
10. Partner status (0=No; 1=Yes)	−0.15^**^	0.06^*^	0.03	0.03	0.10^**^	−0.07^**^	−0.29^**^	−0.06^*^	−0.08^**^	1	
11. Deceased (0=No; 1=Yes)	0.30^**^	−0.18^**^	−0.07^**^	0.06^*^	−0.08^**^	−0.07^*^	−0.12^**^	0.17^**^	0.08^**^	−0.08^**^	1

* *p*<0.05;

** *p*<0.01

Higher education was moderately associated with higher wealth (*r*=0.31). Both markers of SES were significantly and positively associated with perceived control. The bivariate correlations with proportional discrepancy scores for felt age were, however, not statistically significant. Only the association between education and ideal age discrepancies was statistically significant (*r*=−0.06), indicating that those with higher education had smaller ideal age discrepancies. Larger felt age discrepancies were associated with higher levels of perceived control (*r*=0.12), whereas larger ideal age discrepancies were associated with perceived lower control (*r*=−0.08). Respondents reporting larger felt age discrepancies (i.e., feeling younger than their actual age) also reported larger ideal age discrepancies (i.e., wanting to be younger than one’s actual age), but correlations between these two aspects of SA were relatively modest (*r*=0.18). The two aspects of SA were relatively weakly correlated with the other study variables. Perceived control was significantly correlated with all study variables except for smoking cigarettes.

The Cox regression (Table 3 Unadjusted HR) showed that one increasing level of education or one higher quartile of wealth was associated with a 12–19 percent lower risk of elderly mortality. In addition, a 1 standard deviation (SD) unit younger felt age, which refers to feeling approximately 9years younger, was related to a 15 percent lower risk of mortality. On the other hand, a 1 standard deviation unit younger ideal age, which refers to wanting to be approximately 16years younger, was associated with a 12 percent increased mortality risk. There was a relatively strong association between perceived control and elderly mortality: One standard deviation unit increase in perceived control was associated with a 23 percent reduction in mortality. Table 3 also shows the results from the stepwise proportional hazards models. Model 1 indicates that older age and being male were associated with an increased hazard of dying over the follow-up. Model 1 contributed to 11 percent explained variation in survival time. In Model 2, both lower levels of education and wealth were significantly associated with higher mortality risk, irrespective of age and gender. Taking age and gender into account, a one unit increase from lowest SES was associated with a 17 to 18 percent reduction in the hazard of elderly mortality. Adding SES explained an additional 2 percent of the total variation. Ideal and felt age discrepancies were added in Model 3, but the decrease in -2log likelihood was not significant (*χ*^2^=184,59; *df*=6; *p*=0.140) indicating that adding SA did not significantly contribute to improved model fit over and above sociodemographic and SES characteristics. The lack of significant associations with mortality also implied lack of evidence for the mediating role of subjective age in the association between SES and mortality and limits the potentially mediating role of perceived control. In Model 4, lower perceived control was significantly associated with mortality in older adults. Individuals scoring 1 standard deviation higher on the PMS had a 20 percent reduction in mortality risk. Adding perceived control contributed to an additional 1.8 percent explained variation in survival time. The positive associations of higher education and wealth remained, however, statistically significant, suggesting independent associations of SES and perceived control with elderly mortality. The final full model, adjusting for physical health limitations, cigarette smoking, and partner status, yielded the association of education with mortality no longer statistically significant. These core confounders contributed to an additional 5 percent explained variation in longevity conditional upon survival to age 67, summing up to a total of 20.2 percent explained variance. The association of higher levels of wealth and perceived control with reduced elderly mortality remained statistically significant, also after taking important confounders into account.

**Table 3:** Mortality risk in older adults (67+). Bivariate coefficients for and stepwise Cox proportional hazards regression analyses (*N*=1,432). Hazard Ratios (HR) and 95% Confidence Intervals (CI).

Predictors (baseline NorLAG2)	Unadjusted	Model 1 Age, gender	Model 2 SES	Model 3 SA	Model 4 Control	Model 5 Full
	HR (95% CI)	HR (95% CI)	HR (95% CI)	HR (95% CI)	HR (95% CI)	HR (95% CI)
Education (1–3)	0.81 (0.70–0.94)^**^		0.82 (0.70–0.95)^**^	0.82 (0.71–0.97)^*^	0.84 (0.72–0.99)^*^	0.88 (0.75–1.03)
Wealth (1–4)	0.88 (0.81–0.97)^**^		0.83 (0.75–0.92)^***^	0.83 (0.75–0.92)^***^	0.83 (0.75–0.92)^***^	0.82 (0.74–0.91)^**^
Felt age proportional discrepancy (Z- score)	0.85 (0.76–0.96)^**^			0.90 (0.80–1.00)	0.92 (0.82–1.03)	0.96 (0.86–1.07)
Ideal age proportional discrepancy (Z- score)	1.12 (1.01–1.24)^*^			1.03 (0.93–1.14)	1.01 (0.91–1.12)	0.99 (0.89–1.10)
Perceived control (Z-score)	0.72 (0.66–0.79)^***^				0.80 (0.73–0.88)^***^	0.83 (0.75–0.92)^***^
Gender (0=Man; 1=Woman)		0.62 (0.50–0.76)^***^	0.53 (0.43–0.66)^***^	0.54 (0.43–0.687^***^	0.52 (0.42–0.66)^***^	0.43 (0.34–0.54)^***^
Chronological age		1.13 (1.10–1.15)^***^	1.13 (1.11–1.16)^***^	1.13 (1.11–1.15)^***^	1.12 (1.10–1.14)^***^	1.12 (1.09–1.14)^***^
Physical health problems (0=No; 1=Yes)						1.84 (1.47–2.29)^***^
Smoking cigarettes (0=No; 1=Yes)						1.74 (1.34–2.25)^***^
Partner status (0=No; 1=Yes)						0.73 (0.58–0.91)^**^
*-2log likelihood*		*5084.093*	*5055.84*	*5051.95*	*5033.72*	*4978.78*
*∆χ* ^2^			*28.3 (2df)*	*3.88 (2df)*	*18.24 (1df)*	*54.94 (3df)*

* *p*<0.05;

** *p*<0.01;

*** *p*<0.001.

## Discussion

The findings from the current study did not support a mediating role of secondary or primary control strategies in the association between SES and elderly mortality. Rather, lower levels of wealth and perceived control independently predict increased elderly mortality over an 11-year period, after taking core determinants of (inequalities in) life expectancy into account. These findings underline the specific importance of structural conditions (wealth) and suggest the continued importance of (perceived) primary control capacity for longevity also after age 67. Although SA could not account for SES-related variability in life span among older Norwegians, our study showed that feeling younger and wanting to be an age closer to one’s actual age were positively associated with perceived control. This is consistent with the LSD perspective that proposes SA as an important secondary control strategy in older adults. The main findings and study limitations are discussed below.

### The Importance of Accumulated Wealth for Mortality at Older Ages

There is relatively limited research on wealth inequalities in mortality at older ages, partly because of a lack of accurate wealth data from household surveys (Bosworth, 2018). A strength of the current study is the availability of wealth data from the national public registers. Our findings thus contribute to knowledge generated from studies showing persisting wealth inequalities in mortality at older ages, after taking level of education into account (Demakakos et al., 2016; Attanasio and Nielsen, 2020; Zaninotto et al., 2020). Unlike education, wealth measures the accumulation of resources and assets over the life course. Low wealth likely indicates the detrimental effect of the accumulation of disadvantage over the life course and identifies a population at risk (Demakakos et al., 2016). Most research on the association of wealth and mortality stems from the United States, where explanations are geared toward socioeconomic differences in access to healthcare and insurance. Our findings suggest that the importance of wealth for elderly mortality also expands to other national contexts, in this case Norway, a country with an extensive welfare state comprising redistributive policies and universal social protection systems. After age 67, accumulated wealth may be a more accurate marker of SES affecting how older adults are facing challenges associated with advanced age, independent of perceived control. However, wealth and education contributed to explain only 2% of the total variation in elderly mortality, and associations with perceived control were weak. One position that explains this is “age-as-a-leveler” (Dupre, 2007), which suggests that SES inequalities in health are leveled out with increasing age because of mortality selection, in this case higher mortality among those with low education and low wealth before age 67. The relatively weak associations may also reflect the substantial heterogeneity among older persons with low levels of education or wealth, underlining that not all older persons with a low SES can be labeled vulnerable or at risk.

### Psychological Pathways to Elderly Mortality

Similar to previous studies (Bosma et al., 1999; Hall et al., 2010; Chipperfield et al., 2012; Infurna et al., 2013), the current study showed that perceived higher control contributed significantly to reduced elderly mortality over an 11-year follow-up. In terms of LSD theory, these findings support the importance of maintaining or enhancing primary control capacity in older adults (Kunzmann et al., 2002; Daatland and Hansen, 2007; Bercovitz et al., 2019), for example, by emphasizing elements of choice and personal responsibility or through motivational behavioral interventions targeting self-protection strategies (Hall et al., 2010). Such strategies may include goal disengagement, the importance of which is supported by our finding that larger ideal age discrepancies, that is, wanting to be increasingly younger than one’s actual age, are associated with perceived lower control.

Unlike findings from studies including general populations (Bosma et al., 1999; Turiano et al., 2014), our findings did not suggest evidence for people with lower SES being more disadvantaged because of low perceived control or maladaptive SA. This may be due to SES differences in mortality being relatively smaller in older compared with middle-aged people. In addition, we did not find evidence for a direct association of subjective age perceptions with elderly mortality when taking age and gender into account. This may be considered puzzling, given that many studies have shown a robust association between similar measures of older subjective age and higher risk of mortality across adulthood (Kotter-Gruhn et al., 2009; Rippon and Steptoe, 2015; Stephan et al., 2018). The proportional discrepancy score for felt age in our study (0.12) was somewhat smaller than in the Midlife in the United States Survey (MIDUS; 0.16), where a similar measure of SA was used (Stephan et al., 2018). The adjusted HRs for mortality found in our sample were smaller compared to those in MIDUS, 10% versus 18% higher risk of mortality for 1 SD older subjective age. Possible explanations for the limited role of SA in the present study may therefore be related to a smaller sample size or that that the associations of SA with mortality are found to be stronger in countries without an extensive welfare state (Barrett, 2003; Westerhof et al., 2012, 2014).

### Limitations

Our findings are based on a representative sample of community-dwelling older Norwegians and should generalize to the broader population of older non-institutionalized adults (Veenstra et al., 2021). However, greater non-response among those with poor health and lower education may have affected the findings of the current study by underestimating core associations, for example, between education and mortality. Our study had a relatively long timespan and did not include repeated measurements between baseline and the follow-up endpoint. We were therefore not able to include time-varying predictors in studying patterns of elderly mortality, which may be important for detecting short-term associations of SA. In addition, our findings addressed all-cause mortality and we had no data on causes of death. Moreover, the significant contribution of perceived lower control to elderly mortality may also reflect a naturalistic explanation, in that, closer to death, control capacity is reduced and a realistic appraisal of one’s situation with regard to controllability is warranted, which results in lower scores on measures of domain-general perceived control capacity such as the PMS. Our study did not include measures of domain-specific perceived control. People may perceive control in some major domains of life but not in others. Neither did our study include measures of motivational investment, which would have been more in line with Heckausen’s motivational theory of life span development (Heckhausen et al., 2019).

## Conclusion

Perceived control and accumulated wealth contribute independently to mortality in adults aged 67years and older, after taking core determinants of (inequalities in) life expectancy into account. Our findings add to research showing that perceived control is an integral component of healthy aging (Lachman, 2006; Heckhausen et al., 2019) and contribute to the knowledgebase on socioeconomic inequalities in the second half of life by demonstrating persistent wealth inequalities in elderly mortality in a country with an extensive welfare state. Future research is needed to assess in more detail how wealth and perceived control complement each other in contributing to healthy aging and longevity, for example, by longitudinal research including the role of significant life events in the second half of life in different welfare state contexts. Important questions for future research on aging should also include the end of life and provide updated knowledge on how levels of perceived control may contribute to regulate emotions, reduce anxiety, fear of death, and facilitate processes of reconciliation in older adults.

## Data Availability Statement

Publicly available datasets were analyzed in this study. This data can be found here: https://norlag.nsd.no/.

## Ethics Statement

Ethical review and approval was not required for the study on human participants in accordance with the local legislation and institutional requirements. Written informed consent for participation was not required for this study in accordance with the national legislation and the institutional requirements.

## Author Contributions

MV and SD conceived the idea for the study. MV, SD, and GL contributed to its design. MV prepared, analyzed, and interpreted the data and produced the first draft of the manuscript. SD and GL provided feedback on earlier drafts. All authors approved the final version of the manuscript to be published.

## Funding

The research presented here was carried out with financial support from the Research Council of Norway (Grant No. 301958).

## Conflict of Interest

The authors declare that the research was conducted in the absence of any commercial or financial relationships that could be construed as a potential conflict of interest.

## Publisher’s Note

All claims expressed in this article are solely those of the authors and do not necessarily represent those of their affiliated organizations, or those of the publisher, the editors and the reviewers. Any product that may be evaluated in this article, or claim that may be made by its manufacturer, is not guaranteed or endorsed by the publisher.
